# Review of the Japanese records of an endangered grouper, *Epinephelus tukula*, with comments on its population status (Teleostei, Serranidae)

**DOI:** 10.3897/zookeys.772.24374

**Published:** 2018-07-06

**Authors:** Atsunobu Murase, Ryohei Miki, Masaaki Wada, Masahide Itou, Hiroyuki Motomura, Hiroshi Senou

**Affiliations:** 1 Nobeoka Marine Science Station, Field Science Center, University of Miyazaki, 376-6 Akamizu, Nobeoka, Miyazaki 889-0517, Japan; 2 Department of Marine Biology and Environmental Sciences, Faculty of Agriculture, University of Miyazaki, Gakuen-Kibanadai-Nishi, Miyazaki 889-2192, Japan; 3 Interdisciplinary Graduate School of Agriculture and Engineering, University of Miyazaki, 1-1 Gakuen-kibanadai-nishi, Miyazaki 889-2192, Japan; 4 Fisheries Cooperative Association of Iorigawa, 6-188 Iorigawa-nishi, Kadogawa-cho, Higashiusuki-gun, Miyazaki 889-0605, Japan; 5 718 Kataura, Kasasa, Minamisatsuma, Kagoshima 897-1301, Japan; 6 The Kagoshima University Museum, 1-21-30 Korimoto, Kagoshima 890-0065, Japan; 7 Kanagawa Prefectural Museum of Natural History, 499 Iryuda, Odawara-shi, Kanagawa 250-0031, Japan

**Keywords:** Potato Grouper, range, Red List, threatened species, voucher

## Abstract

The Potato Grouper, *Epinephelus tukula*, is relatively rare worldwide. Records from the northernmost part of its range (Japan) have been few, resulting in a “Critically Endangered” listing on the Red List for Japan. The Japanese records were revised by examining literature, new specimens, photographs, and the internet, and a continuous distribution pattern from the tropical Ryukyu Islands (including adult individuals) to temperate regions affected by the Kuroshio Current were delineated; this suggests the species inhabits tropical Japan and can spread to temperate regions via the warm current. The species possibly reproduces in Japanese waters but further reproductive ecology research is required.

## Introduction

Fishes of the subfamily Epinephelinae (Perciformes, Serranidae), known as groupers, are distributed mainly in tropical and subtropical waters of the world; they are carnivorous, feeding on invertebrates and fishes, occupy important positions in reef ecosystems, and are an important fishery group with high commercial value (Craig et al. 2011). Many grouper fisheries worldwide are in decline but those in the western Pacific are generally expanding. Few grouper fisheries are adequately and consistently monitored, and specific data on population trends and size frequency distribution are needed (Craig et al. 2011). Japanese waters represent the northernmost distribution of many grouper species (Randall and Heemstra 1991; Craig et al. 2011; Senou 2013) and, considering their high economic value and population status in the region, seven species of Epinephelinae have been ranked as threatened in the Japanese Red List for marine organisms (Ministry of the Environment 2017).

The Potato Grouper, *Epinephelus tukula* Morgans, 1959, attaining 2 m in total length is one of the largest species in the family, and is sparsely distributed in the Indo–Pacific region, from the Red Sea to South Africa, Comoro Islands, the Seychelles, Oman, Pakistan, India, Sri Lanka, Maldives and Laccadives in the Indian Ocean, from Japan to the South China Sea in the northern sphere of the Pacific Ocean, and around the northern area of Australia in the southern sphere of the Pacific; it appears to be naturally uncommon throughout its range (Heemstra and Randall 1993; Craig et al. 2011). The IUCN Red List ranked the species as Least Concern (LC) despite the list summarizing that the species is uncommon and vulnerable to capture, and concluding that the species should be re-evaluated with more information on its biology and with respect to the impacts of fisheries (Fennessy et al. 2008). On the other hand, the Japanese Red List for marine organisms ranks the species as Critically Endangered (CR) based on the criterion that the number of mature individuals is continuously decreasing (Ministry of the Environment 2017). However, the evidence for this ranking was four earlier reports (including two faunal surveys without any vouchers) and unpublished photographic records (Katayama 1975; Masuda et al. 1975; Ichikawa et al. 1992; Hirata et al. 1996), and market observations (H. Senou personal communication) that resulted in no Japanese records of the species for last 40 years. Recent ichthyofaunal surveys by the authors along the coasts of south-east Kyushu in southern Japan, where historically few fish faunal studies have been conducted, found new specimens and photographs of *E. tukula*. These new records are detailed herein, along with a review of the distribution records and size information of *E. tukula* in Japan in order to re-evaluate the population status at the northern extent of its range.

## Materials and methods

Counts and measurements followed Randall and Heemstra (1991) but predorsal- and preanal-fin lengths are the length from the tip of the snout to the origins of the dorsal- and anal-fins. Measurements were made with needle-point calipers to the nearest 0.1 mm. New specimens of *Epinephelus tukula* from Japanese waters were captured by various types of fishing gear at several sites on the south and east coasts of Kyushu (Kagoshima and Miyazaki prefectures), southern Japan, during 2012–2017. These specimens were fixed in 10% formalin and subsequently preserved in 70% ethanol. All specimens were deposited in the ichthyological collection of the Kagoshima University Museum (KAUM–I.) or the Kanagawa Prefectural Museum of Natural History (KPM-NI). Color photographs of a specimen in both live and fresh condition, and of individuals observed in fish markets, were deposited in the image database of the Kanagawa Prefectural Museum of Natural History (KPM-NR). The registration numbers of KPM are expressed in seven figures including a preceding “0” (e.g. KPM-NR0180045) on the museum database. Information on the species with images (photographs or videos) on web sites were cited as the distribution records. The exact meaning of a Japanese term “taicho”, translated literally as body length, stated on web sites is ambiguous: it can be standard or total lengths. In this case, the size information was recorded as total length with “at least” (e.g. at least 120 cm in total length). Size of fish individual on a video of a web site was estimated as “at least 100 cm in total length” based on the fish size being almost same length as height of the angler filmed with the fish.

Specimens examined (6 individuals from south and east coasts of Kyushu)– Miyazaki Prefecture: KPM-NI 42540 (KPM-NR 180045A–H), 544.0 mm standard length (SL), Kadogawa Bay, Kadogawa Town, Higashiusuki-gun, 32°28'14.498"N, 131°40'47.014"E, 28 Jan. 2017, captured by local fisherman with set net; Kagoshima Prefecture: KAUM–I. 46905 (KPM-NR 185007A–C), 499.2 mm SL, off Kouzakiyama, Kataura, Kasasa Town, Minamisatsuma City, 31°26'N, 130°10'E, 28 Apr. 2012, purchased by Masahide Itou which captured by local fisherman with gill net; KAUM–I. 53306, 502.0 mm SL, off Kunigami, Nishino-omote City, Tanega-shima island, 30°49'N, 131°01'E, 26 Feb. 2013, purchased by Mayumi Takayama which captured by local fisherman with spear; KAUM–I. 62330, 374.0 mm SL, Nakayama Fishing Port, Noma, Nakatane Town, Kumage-gun, Tanega-shima island, 30°31'40"N, 130°59'35"E, 6 June 2014, captured by Koichi Kaburagi with hook and line; KAUM–I. 70614 (KPM-NR 185010A–D), 238.5 mm SL, 2 km off Koura, Kataura, Kasasa Town, Minamisatsuma City, 31°25'N, 130°12'E, 21 Feb. 2015, captured by Masahide Itou with set net; KAUM–I. 74330, 375.5 mm SL, off Ibusuki City, Kagoshima Bay, 31°14'N, 130°41'E, 22 Jun. 2015, purchased by Harutaka Hata which captured by local fisherman with hook and line.

Photographs examined (5 individuals, from south and east coasts of Kyushu)– Miyazaki Prefecture: KPM-NR 179394A–C, ca 100 cm total length (TL), landed at Meitsu Fishing Port, Nango Town, Nichinan City, 17 May 2012, photographed by Ryohei Miki; KPM-NR 179395A–D, ca 50 cm TL, same locality and photographer as KPM-NR 179394, 17 Nov. 2012; Kagoshima Prefecture: KPM-NR 77829A, B, young individual, landed at Kataura Fishing Port, Kasasa Town, Minamisatsuma City, 17 May 2012, photographed by Masahide Itou; KPM-NR 185008A–D, young individual, same locality and photographer as KPM-NR 77829, 23 Aug. 2014; KPM-NR 185009A–D, young individual, same locality and photographer as KPM-NR 77829, 3 Dec. 2014.

## Description

Counts and measurements of *Epinephelus tukula* captured from the coasts of Kyushu are shown in Table 1. Body shape elongated-oval; snout slightly pointed, its length longer than orbit diameter; interorbital space slightly convex; dorsal head profile straight or slightly concave; posterior nostril usually 1.2–1.4 times larger than anterior nostril but anterior one 1.1–1.6 times larger than posterior in two individuals (238.5 and 374.0 mm SL); maxilla extending slightly beyond a vertical at posterior edge of orbit; four or six rows of teeth on tip of lower jaw; three rows of teeth on mid-side of lower jaw; serrae at corner of preopercle moderately enlarged; margins of subopercle and interopercle smooth; opercle with three spines but lowest obscure and blunt; third or fourth (usually fourth) dorsal-fin spine longest; membranes of spinous dorsal fin incised; third anal-fin spine 1.3–1.5 times longer than second; caudal fin slightly rounded; posterior edge of pectoral fin not reaching vertical at anal-fin origin, reaching beneath 7–8^th^ dorsal-fin spine bases; scales on body mostly ctenoid, but cycloid on anterodorsal, ventral and thoracic areas of body; auxiliary scales surrounding regular scales in almost all scaled regions; small scales crowded in triangular patch on upper side of maxilla; lateral-line scales countable only in smallest specimen because some lateral-line pores very obscure or broken in the five larger specimens.

**Table 1.** Counts and measurements of *Epinephelus tukula* specimens from Kyushu, southern Japan.

Locality and voucher specimen	Southwestern Kyushu		Southern Kyushu	Tanega-shima island		Eastern Kyushu
	KAUM–I. 46905	KAUM–I. 70614	KAUM–I. 74330	KAUM–I. 53306	KAUM–I. 62330	KPM-NI 42540
Standard length (SL, mm)	499.2	238.5	375.5	502.0	374.0	544.0
Total length (mm)	611.0	298.9	468.2	612.1	461.3	668.0
Counts						
Dorsal-fin rays	XI, 15	XI, 15	XI, 15	XI, 15	XI, 15	XI, 15
Anal-fin rays	III, 8	III, 8	III, 8	III, 8	III, 8	III, 8
Pectoral-fin rays	20	20	20	19	20	20
Pelvic-fin rays	I, 5	I, 5	I, 5	I, 5	I, 5	I, 5
Longitudinal scale rows	111	121	110	117	109	122
Lateral-line scales	–	63	–	–	–	–
Upper gill rakers	10	10	9	9	8	11
Lower gill rakers	15	15	15	15	16	21
Total gill rakers	25	25	24	24	24	32
Measurements (% SL)						
Head length	39.5	40.0	40.5	39.3	39.9	41.3
Snout length	11.5	9.6	11.2	11.4	11.0	11.9
Upper-jaw length	20.0	18.9	19.6	19.5	20.0	19.4
Interorbital width	7.2	4.9	6.5	6.7	6.6	7.6
Orbit diameter	4.2	5.5	4.8	4.4	5.1	4.1
Body depth	30.4	32.8	33.8	30.6	35.3	31.9
Caudal peduncle length	16.9	15.9	16.2	15.6	16.5	16.1
Caudal peduncle depth	12.3	12.1	12.7	12.3	12.6	11.7
Predorsal length	36.9	36.6	38.4	36.1	37.0	37.2
Preanal-fin length	70.5	70.1	69.7	72.1	69.7	73.2
1^st^ dorsal-fin spine length	4.1	5.5	4.4	4.1	4.5	4.3
2^nd^ dorsal-fin spine length	8.9	9.5	8.5	8.1	9.1	8.5
3^rd^ dorsal-fin spine length	9.9	10.6	9.7	9.7	11.2	10.7
4^th^ dorsal-fin spine length	10.0	10.8	9.8	10.1	11.3	10.6
5^th^ dorsal-fin spine length	9.3	10.6	9.2	9.7	11.0	10.4
Last dorsal-fin spine length	9.3	9.8	9.6	8.7	9.2	8.2
Length of longest dorsal-fin ray	14.5	16.1	15.3	13.9	15.3	13.9
1^st^ anal-fin spine length	2.9	4.1	4.2	2.5	3.0	3.1
2^nd^ anal-fin spine length	5.3	8.0	6.8	5.3	5.5	5.4
3^rd^ anal-fin spine length	7.9	8.2	9.5	7.3	6.9	7.8
Length of longest anal-fin ray	16.4	18.3	18.5	16.8	19.5	15.5
Pectoral-fin length	18.6	20.9	21.0	19.4	20.6	19.0
Pelvic-fin length	16.2	18.1	17.4	16.7	18.3	16.0

**Figure 1.**
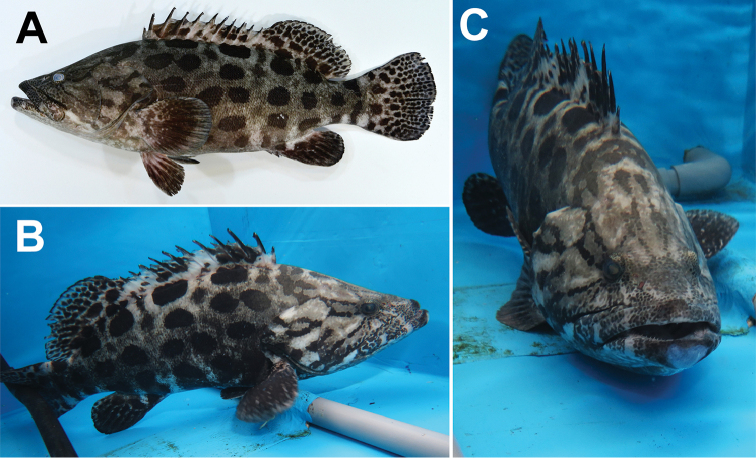
Color images of *Epinephelus tukula* (KPM-NI 42540, 544.0 mm SL) captured at Kadogawa Bay, Miyazaki Prefecture, eastern coast of Kyushu, southern Japan: **A** lateral view of fresh specimen (photo number KPM-NR 180045A, photo by H. Senou) **B** lateral view of live condition in a market tank (KPM-NR 180045E, R. Miki) **C** frontal view of live condition in a market tank (KPM-NR 180045D, R. Miki).

Base color of head and body light gray to dark brown in fresh condition (Fig. 1A); dark brown oval or circular blotches on body appearing to form several columns parallel with body axis, blotches mostly larger than eye but subequal to or smaller than eye in smaller specimens; head blotches smaller than body blotches; very small dark brown spots around snout and upper jaw regions; irregular narrow spots radiating from posterior edge of eye to almost posterior edge of preopercle; larger irregular spots on opercular and subopercular regions; dark spots on soft portion of each fin, smaller distally, but several dark blotches on spinous portion of dorsal fin; soft portion of anal, pectoral and pelvic fins darker in largest specimen, with spots obscure. Live coloration similar to fresh (Fig. 1B, C). In preservative, darker than in fresh condition but spots remaining visible.

## Distribution

**Figure 2.**
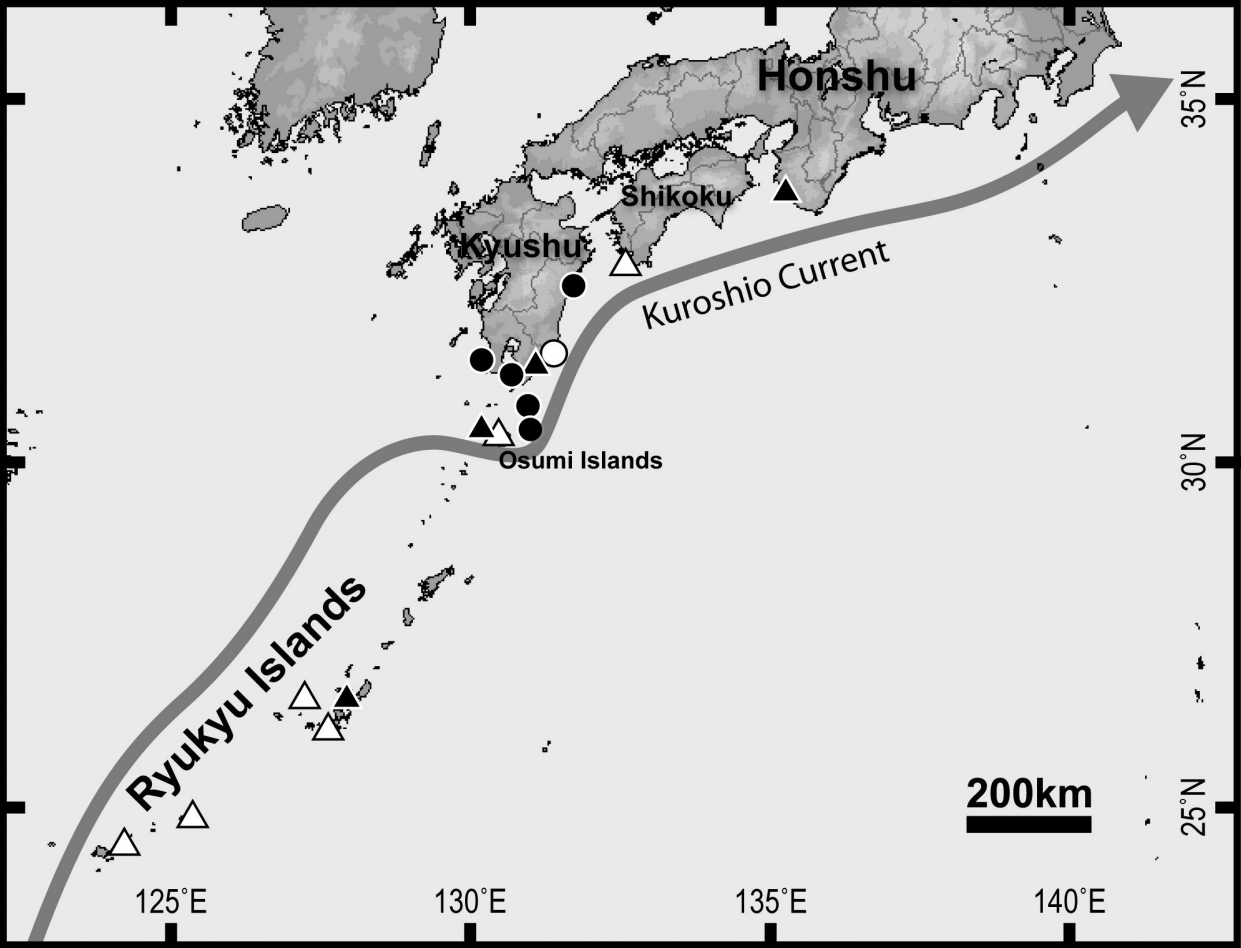
Distribution map of *Epinephelus tukula* in Japanese waters. Key: solid circle, specimen records from the present study; open circle, photo records from the present study; solid triangle, voucher (specimen or photo) records from the past studies; open triangle, literature records without any vouchers or web records. Arrow showing the course of Kuroshio Current.

The distribution records of *Epinephelus tukula* from Japanese waters including new material and web information are summarized in Table 2, and each locality is mapped in Fig. 2. Examination of these records show a continuous distribution along the tropical Ryukyu Islands and Pacific coasts of southern Japan, i.e., the region significantly influenced by the warm Kuroshio Current. Size information is also summarized in Table 2 with specimens of approximately 30–134 cm TL recorded from Japanese waters. Most individuals recorded north of the Ryukyu Islands were young/immature but one individual (ca. 100 cm TL) recorded from Nichinan City probably exceeded the smallest maturity size of females (about 90 cm SL: Yeh et al. 2003). On the other hand, at least three individuals recorded from the Ryukyu Islands were larger than 100 cm TL and therefore of mature size.

**Table 2.** Distribution records of *Epinephelus tukula* from Japanese waters based on literature, voucher and web sources.

Locality			Occurrence season		Size information		Source	
Region	Prefecture	City, town or island	Month	Season	Total length	Life stage	Literature	Voucher
Central Honshu	Wakayama	Minabe Town	Nov	Autumn	ca 30.0 cm	Young	Masuda et al. (1975)	MS
Shikoku	Kochi	Kashiwa-jima island	–	Autumn	ca 70.0 cm	Young	Hirata et al. (1996)	None
Kyushu	Miyazaki	Kadogawa Town	Jan	Winter	66.8 cm	Young	Present study	MS, MP
		Nichinan City	May, Nov	Spring, Autumn	ca 50–100 cm	Young/mature	Present study	MP
	Kagoshima	Minamisatsuma City	Feb, Apr	Winter, Spring	29.9–61.1 cm	Young	Present study	MS
			May, Aug, Dec	Spring–Winter	–	Young	Present study	MP
		Uchinoura Bay	Feb, Nov	Winter, Autumn	–	–	Hata (2018)	P
		Ibusuki City	Jun	Summer	46.8 cm	Young	Present study	MS
Osumi Islands	Kagoshima	Tanega-shima island	Feb, Jun	Winter, Summer	46.1–61.2 cm	Young	Present study	MS
		Yaku-shima island	–	–	–	–	Ichikawa et al. (1992)	None
		Kuchinoerabu-jima island	Nov	Autumn	–	Maybe mature	Kimura et al. (2017)	P
Ryukyu Islands	Okinawa	Okinawa Islands	–	–	33.0 cm	Young	Katayama (1975)	MS
		Itoman City	–	–	–	–	Masuda et al. (1975)	None
		Aguni-jima island	–	–	At least 120 cm	Mature	Web1	P
		Miyako-jima island	–	–	At least 100 cm	Mature	Web2	V
		Yaeyama Islands	–	–	134 cm	Mature	Akita et al. (2016)	None

Literature (web source): Web1, http://okinawa-zukan.com/f_Epinephelus_tukula.htm; Web2, https://ibowbow.com/us/video/1CigOabmgY0Voucher abbreviations: MP, museum photos; MS, museum specimens; P, photos; V, video

## Discussion

The six specimens collected from Miyazaki and Kagoshima prefectures corresponded well with the descriptions and images of *Epinephelus tukula* in Katayama (1975), Masuda et al. (1975), Randall and Heemstra (1991), Heemstra and Randall (1993), Craig et al. (2011), and Senou (2013) in having the following diagnostic characters: dorsal-fin rays XI, 15; anal-fin rays III, 8; pectoral-fin rays 19–20; scales on body ctenoid but cycloid scales on anterodorsal, ventral and thoracic areas of body; auxiliary scales surrounding regular scales in almost all scaled regions; gill rakers 8–10 + 15–16 = 24 or 25 (except the largest specimen); interorbital space slightly convex; membranes of spinous dorsal-fin incised; caudal fin slightly rounded; base color of head and body light gray to dark brown with dark brown to black spots, most larger than eye; smaller spots on head than body; irregular narrow spots radiating from posterior edge of eye. The range in number of longitudinal scale rows in the present study (109–122) was slightly less than that of earlier studies (113–130: Randall and Heemstra 1991; Heemstra and Randall 1993) and the number of gill rakers of the largest specimen in this study (11 + 21) exceeded that recorded in an earlier study (8–10 + 15–18: Heemstra and Randall 1993). Because Randall and Heemstra (1991) redescribed *E. tukula* based on nine specimens from the Indian Ocean and Eastern Australia, the differences in numbers of scale rows between the earlier and present studies may be caused by the small sample sizes and regional variations. Regarding the number of gill rakers, Katayama (1975) recorded 9 +19 in his description of the *E. tukula* from the Okinawa Islands, which is also outside the range in Randall and Heemstra (1991). The Japanese population might have a tendency for higher numbers of gill rakers than in other regions for unknown reasons.

**Figure 3.**
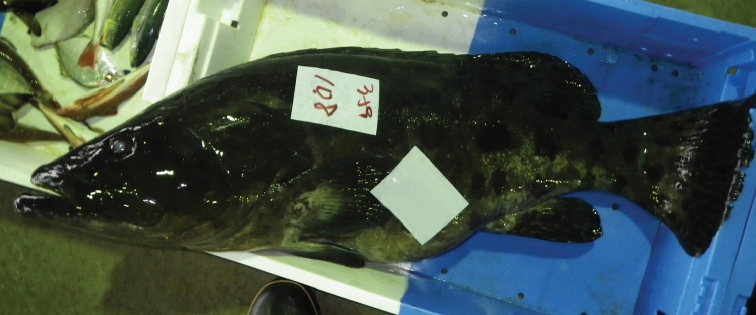
An adult individual of *Epinephelus tukula* (ca 100 cm TL, photo number KPM-NR 179394A) landed at Meitsu Fishing Port, Nichinan City, Miyazaki Prefecture, eastern coast of Kyushu, southern Japan. Photograph R. Miki, 17 May 2012.

The results of the present study revealed that the distribution range of *Epinephelus tukula* in Japan is continuous from the Ryukyu Islands located in a tropical/subtropical region to the Pacific coasts of the Japanese mainland located in the region of the warm Kuroshio Current (Fig. 2, Table 2). Of 66 species of Japanese groupers [tribe Epinephelini, sensu Senou (2013)], 35 species (including *E. tukula*) are continuously distributed from the Ryukyu Islands to the Pacific coast of Japanese mainland, and most of these are also known from elsewhere in the Indo-Pacific region (Senou 2013; Fujiwara et al. 2015). The Kuroshio Current, which flows from the eastern part of the Philippines to the Pacific coast of Japan, transports larvae and juveniles of many tropical fishes and invertebrates to temperate regions of Japan (Senou et al. 2006; Sekiguchi 2009). These facts suggests that the tropical groupers (the species which are mainly distributed in tropical zone) known from Japanese waters are distributed not only in the tropical Ryukyu Islands but can disperse northeastward to warm temperate regions by the Kuroshio Current. Furthermore, fingerling production of some species of tropical groupers occurs in the Ryukyu Islands (Okinawa Prefecture 2013), and a spawning aggregation in a grouper species has been reported (Nanami et al. 2017). These facts suggest that most of the tropical groupers occurring in Japan inhabit and reproduce in the Ryukyu Islands. The present study documents records of adult individuals of *E. tukula* from the Ryukyu Islands, indicating that this species is also most likely to reproduce in this region. On the other hand, only a single adult specimen of the species (KPM-NR 179394: Fig. 3) has been recorded from the temperate Japanese mainland at Nichinan City, Miyazaki Prefecture. Some studies have recorded adult individuals of tropical fish species from temperate areas of Japan and individuals may have been transported by the Kuroshio Current after maturing in their core distributional area (Motomura et al. 2007a; Ogihara et al. 2010; Itou et al. 2011; Senou et al. 2013). However, *E. tukula* is a large, territorial, epibenthic species (Craig et al. 2011), and may not migrate between regions (i.e. from tropical to temperate) after maturation. Motomura et al. (2007b) also documented a similar case in a congener, *Epinephelus amblycephalus*. Therefore, the adult individual reported from Nichinan City in the present study may have matured after settlement or hatched in the region. In conclusion, Japanese populations of *E. tukula* possibly reproduces in both the Ryukyu Islands and the warm temperate region of Japan that is affected by Kuroshio Current. On account of the facts that *E. tukula* has high commercial value and is vulnerable to fishing pressure, and that the species possibly reproduces in Japan, its Japanese Red List for marine organisms ranking as CR has been effective in terms of giving attention to the status of the species’ northernmost population, including both the tropical Ryukyu Islands and the temperate region further north. Further delineation of the *E. tukula* populations in Japan can be determined by future studies on size frequency distributions and reproductive biology.

## References

[B1] Akita Y Ohta I Ebisawa A Uehara M (2016) Estimation of the fish catches of coastal species of the Yaeyama Islands. Fauna Ryukyuana 31: 13–27. [In Japanese with English abstract]

[B2] Craig MT Sadovy de Mitcheson YJ Heemstra PC (2011) Groupers of the World. A Field and Market Guide. NISC (Pty) Ltd, Grahamstown, 356 pp.

[B3] Fennessy S Pollard D Myers R (2008) *Epinephelus tukula* The IUCN Red List of Threatened Species 2008: e.T132773A3447657. 10.2305/IUCN.UK.2008.RLTS.T132773A3447657.en [Accessed 3 February 2018]

[B4] Fujiwara K Takayama M Sakurai Y Motomura H (2015) Records of *Epinephelus bleekeri* (Perciformes: Serranidae) from Japan, with notes on distributional implications. Taxa, Proceedings of the Japanese Society of Systematic Zoology 39: 40–46. [In Japanese with English abstract]

[B5] Heemstra PC Randall JE (1993) FAO Species Catalog. Vol. 16. Groupers of the World (Family Serranidae, Subfamily Epinephelinae) An Annotated and Illustrated Catalogue of the Grouper, Rockcod, Hind, Coral Grouper and Lyretail Species Known to Date. FAO Fisheries Synopsis No. 125. FAO, Rome, 382 pp.

[B6] Hirata T Yamakawa T Iwata A Manabe S Hiramatsu W Ohnishi N (1996) Fish fauna of Kashiwa-jima Island, Kochi Prefecture, Japan. Bulletin of Marine Sciences and Fisheries, Kochi University 16: 1–177. [In Japanese with English abstract]

[B7] Ichikawa S Sunakawa S Matsumoto T (1992) A general view of fishes of Yaku-shima Island [original title in Japanese: Yakushima san gyorui no gaikan]. In: Team for Marine Organism Survey in Inshore of Yaku-shima Island [Yakushima engan kaiyou seibutsu Chousadan] (Eds) Report on scientific survey of marine organisms from inshore of Yaku-shima Island [Yakushima engan kaiyou seibutsu gakujyutsu chousa houkokusyo], 19–46. [In Japanese]

[B8] Itou M Matsunuma M Iwatsubo H Motomura H (2011) Northernmost record of a siganid fish, *Siganus guttatus* (Bloch, 1787), from the Kagoshima mainland, southern Kyushu, Japan. Nature of Kagoshima 37: 161–164. [In Japanese]

[B9] Katayama M (1975) Serranid fishes of the Okinawa Islands (III). Bulletin of the Faculty of Education, Yamaguchi University 25: 161–178.

[B10] Kimura Y Hibino Y Miki R Minetoma T Koeda K (2017) Field Guide to Fishes of Kuchinoerabu-jima Island in the Osumi Group, Kagoshima, southern Japan. The Kagoshima University Museum, Kagoshima, 200 pp. [In Japanese]

[B11] Hata H (2018) Serranidae. In: Koeda K Hata H Yamada M Motomura H (Eds) Field guide to fishes landed at Uchinoura Fishing Port, Kagoshima, Japan. The Kagoshima University Museum, Kagoshima, 186–198. [In Japanese]

[B12] Masuda H Araga C Yoshino T (1975) Coastal Fishes of Southern Japan. Tokai University Press, Tokyo, 378 pp.

[B13] Ministry of the Environment (2017) Japanese Red List for marine organisms [original title in Japanese: Kankyosho ban kaiyo seibutsu Red List no kohyo ni tsuite]. http://www.env.go.jp/press/103813.html [accessed on 11 January 2018]

[B14] Motomura H Ito M Takayama M Haraguchi Y Matsunuma M (2007a) Second Japanese record of a threadfin, *Eleutheronema rhadinum* (Perciformes, Polynemidae), with distributional implications. Biogeography 9: 7–11.

[B15] Motomura H Ito M Ikeda H Endo H Matsunuma M Hatooka M (2007b) Review of Japanese records of a grouper, *Epinephelus amblycephalus* (Perciformes, Serranidae), with new specimens from Kagoshima and Wakayama. Biogeography 9: 49–56.

[B16] Nanami​ A Sato T Kawabata Y Okuyama Y (2017) Spawning aggregation of white-streaked grouper *Epinephelus ongus*: spatial distribution and annual variation in the fish density within a spawning ground. Peer J 5: e3000. 10.7717/peerj.3000 PMC5312569 28229026

[B17] Ogihara G Yoshida T Ito M Yamashita M Sakurai Y Motomura H (2010) Record of *Bolbometopon muricatum* (Labroidei: Scaridae) from Kasasa, Kagoshima, southern Kyushu, Japan. Nature of Kagoshima 36: 43–47. [In Japanese]

[B18] Okinawa Prefecture (2013) Introduction to fishery species of seedling production [original title in Japanese: Shubyo Seisan Taishosyu no Shokai]. http://www.pref.okinawa.jp/site/norin/saibai/saisenituite/taisyousyu.html. [accessed on10 January 2018]

[B19] Randall JE Heemstra PC (1991) Revision of Indo-Pacific groupers (Perciformes: Serranidae: Epinephelinae), with descriptions of five new species. Indo-Pacific Fishes 20: 1–332.

[B20] Sekiguchi H (2009) Dispersal of planktonic larvae of marine benthic invertebrates and its implication. Bulletin on Coastal Oceanography 46: 85–100. [In Japanese with English abstract]

[B21] Senou H (2013) Serranidae. In: Nakabo T (Ed.) Fishes of Japan with pictorial keys to the species, third edition. Tokai University Press, Hadano, 757–802, 1960–1972. [In Japanese]

[B22] Senou H Matsuura K Shinohara G (2006) Checklist of fishes in the Sagami Sea with zoogeographical comments on shallow water fishes occurring along the coastlines under the influence of the Kuroshio Current. Memoirs of the National Science Museum 41: 389–542.

[B23] Senou H Mishiku A Ito M Motomura H (2013) First records of a rare unicornfish, *Naso mcdadei* (Perciformes : Acanthuridae), from Japan, with notes on biogeographical implications for the species. Bulletin of Kanagawa Prefectural Museum (Natural Science) 42: 91–96. [In Japanese with English abstract]

[B24] Yeh S-L Dai Q-C Chu Y-T Kuo C-M Ting Y-Y Chang C-F (2003) Induced sex change, spawning and larviculture of potato grouper, *Epinephelus tukula*. Aquaculture 228: 371–381. 10.1016/S0044-8486(03)00316-8

